# Ocular Lymphatics in Health and Disease

**DOI:** 10.3390/diagnostics16101416

**Published:** 2026-05-07

**Authors:** Nikolaos Anagnostou, Chris Kalogeropoulos, Panagiotis Kanavaros, Alejandra de-la-Torre, Sofia Androudi, Panos Kakoulidis, Rafael Tierradentro-Alape, Dimitrios Kalogeropoulos

**Affiliations:** 1 School of Health Sciences, Faculty of Medicine, University of Ioannina, 45500 Ioannina, Greece; nikosanagnostou2000@gmail.com; 2 Department of Ophthalmology, School of Health Sciences, Faculty of Medicine, University of Ioannina, 45500 Ioannina, Greece; kalogch@otenet.gr; 3 Department of Anatomy-Histology-Embryology, School of Health Sciences, Faculty of Medicine, University of Ioannina, 45500 Ioannina, Greece; pkanavar@uoi.gr; 4 Neuroscience Research Group (NEUROS), Neurovitae Center for Neuroscience, School of Medicine and Health Sciences, Institute of Translational Medicine (IMT), Universidad del Rosario, Bogotá 111221, Colombia; alejadelatorre@yahoo.com (A.d.-l.-T.); raeltierr@gmail.com (R.T.-A.); 5 Department of Ophthalmology, University Hospital of Larissa, 41334 Larissa, Greece; androudi@otenet.gr; 6 Department of Informatics and Telecommunications, School of Science, National and Kapodistrian University of Athens (NKUA), 15701 Athens, Greece; pkakoulidis@di.uoa.gr; 7 Department of Ophthalmology, Stoke Mandeville Hospital, Buckinghamshire Healthcare NHS Foundation Trust, Mandeville Rd, Aylesbury HP21 8AL, UK

**Keywords:** ocular lymphatics, lymphangiogenesis, aqueous humor drainage, ocular fluid homeostasis

## Abstract

The scope of the present study is to conduct a comprehensive review of the anatomy, function, major pathological conditions and clinical significance of ocular lymphatic vessels. In recent years, it has become evident that ocular lymphatics play a major role in the pathogenesis and progression of ocular disorders. Therefore, we delved into this emerging field and described newly proposed mechanisms that may explain the involvement of ocular lymphatics in healthy and pathological states. Lymphatics are present in a plethora of ocular tissues primarily under pathological conditions, including limbal stroma, optic nerve, ocular muscles, lacrimal glands and sclera. The conjunctiva contains an extensive lymphatic network, whereas the cornea and retina are typically devoid of lymphatic vessels under physiological conditions. Inflammatory conditions can give birth to nascent lymphatic vessel sprouting. Growing evidence emphasizes the role of ocular lymphatics in glaucoma pathogenesis, suggesting a previously unknown aqueous humor drainage pathway mediated by lymphatic contribution. This review is based mainly on evidence from animal and experimental models, as human clinical data remain limited; therefore, caution is warranted when translating these findings into clinical practice. By gaining insight into the mechanisms and the clinical significance of eye lymphatics, this review aims to address novel insights for future research and treatment of eye diseases, as well as to highlight a misconception that has persisted for decades in ophthalmology.

## 1. Introduction

Historically, the eye has been deemed an organ devoid of lymphatic vessels, unlike most human body tissues [1,2]. However, in recent years, it has become evident that lymphatic vessels not only exist in ocular tissues but also play a major role in ocular diseases and pathologies. The long-standing belief that lymphatic structures exist in ocular tissues such as the eyelids, lacrimal glands, optic nerve sheath, conjunctiva, corneal limbus and extraocular muscles has been substantiated by modern technological advancements [3,4,5]. Traditionally, the cornea has been recognized as devoid of lymphatic vessels, a phenomenon termed “corneal (lymph)angiogenic privilege” [6]. Evolving evidence shows that Schlemm’s canal acts as a lymphatic vessel, while other studies highlight the importance of lymphangiogenesis under inflammatory conditions in previously thought lymphatic-free tissues such as the cornea [7]. The conjunctiva possesses an abundance of lymphatic vessels, whereas the cornea and retina are devoid of them [8]. Notably, a wide range of diseases have been associated with lymphatic involvement such as inflammatory diseases, ocular tumors and metastasis, glaucoma, uveitis and systematic disease manifestations [1]. Remarkably, angiogenesis and lymphangiogenesis can coexist and are commonly induced by trauma, chemical burns and infections [9]. Advancing knowledge of lymphatics and lymphangiogenesis in the eye will facilitate the development of innovative therapeutic approaches aimed at preventing vision loss in ocular diseases.

The term “lymph” originates from the ancient Greek *νύμφη* (Nymph), referring to a young girl, being or spirit associated with clear streams, and/or the Latin *lympha*, denoting an ancient Roman deity of fresh water [2].

The lymphatic system plays a pivotal role in tissue homeostasis, absorption of dietary fat and fat-soluble vitamins and immune regulation by draining interstitial fluids, metabolites, and immune cells into systemic circulation. Within the tissue space, extravasated fluid, cells, proteins, lipids, and large molecules—collectively referred to as “lymph fluid”—are reabsorbed by lymphatic capillaries and subsequently delivered back into systemic circulation collecting lymphatic vessels [10,11]. Lymphatic capillaries are responsible for the delivery and removal of cellular debris, bacteria, viruses, and immune cells from the lymph-to-lymph nodes. They are also responsible for the trafficking of B cells, T cells, and presenting cells (APCs) from peripheral tissues to lymph nodes to initiate the immune response [12,13].

Although the role of ocular lymphatics has been investigated in previous studies dating back more than a decade, substantial progress in imaging technologies, molecular identification, and also the emerging concept of the so-called ocular glymphatic system have improved our understanding. More recent reviews focus on anatomical description and lymphangiogenesis. However, it appears that there is still no integrated perspective that associates lymphatic and glymphatic pathways with more clinical and diagnostic applications. Our review attempts to approach this gap by (i) summarizing the current knowledge on lymphatic and glymphatic ocular systems, (ii) underlining their clinical and diagnostic implications, and (iii) emphasizing unresolved controversies and translational restrictions. Thus, this work offers an updated and clinically focused framework that reflects current clinical needs and identifies directions for future research. It is important to highlight though that this review relies primarily on findings deriving from animal and experimental studies, as human clinical evidence remains scarce; therefore, these results should be interpreted with caution when applied to clinical practice.

## 2. Materials and Methods

A strategic search was conducted via the PubMed/MEDLINE database. The search encompassed studies published from 1983 to 2025, utilizing primary keywords and Boolean operators including: “ocular lymphatics,” “eye,” “lymphangiogenesis,” and “ocular lymphatic drainage.” Articles were screened for relevance, with a focus on peer-reviewed original research and seminal reviews. Inclusion criteria prioritized studies investigating lymphatic structures within the eye and their role in ocular homeostasis, while exclusion criteria filtered out studies that were redundant, lacked clinical or physiological correlation, or focused on non-ocular lymphatic systems. Unless otherwise specified, the majority of mechanistic insights analyzed throughout review arise from studies based on animal models, particularly murine and rabbit studies. Where available, findings from human tissues or clinical studies are explicitly indicated. This distinction is crucial, as anatomical and physiological variations between different species may affect the applicability and translational relevance of these results.

## 3. Normal Functional Anatomy and Histology of the Lymphatic System

### 3.1. General Aspects of the Lymphatic System

In terms of anatomy, the lymphatic system differs from the blood system in various ways. Firstly, lymphatic vessels function as one-way channels rather than as a closed circuit. Secondly, lymphatic endothelial cells (LECs) lack tight junctions, pericytes and a continuous basement membrane, features that are characteristic of blood circulatory endothelial cells [14]. In terms of morphology, lymphatic capillaries feature a broader and more irregular lumen in comparison to blood capillaries, while a hallmark characteristic of lymphatic capillaries is the presence of anchoring filaments that link lymphatic endothelial cells to the surrounding extracellular matrix [12]. In response to elevated interstitial fluid pressure, anchoring filaments exert tension on intercellular junctions and therefore facilitate the entry of interstitial fluid and cells into the capillary lumen. Upon fluid uptake, these junctions close, effectively preventing the retrograde movement of fluid back into the interstitial space [12,15]. Unlike lymphatic capillaries, collecting lymphatic vessels are composed of spindle-shaped endothelial cells that possess a fully developed basement membrane and are enveloped by one or two layers of smooth muscle cells [16,17,18]. Furthermore, they contain intraluminal valve structures that ensure the unidirectional flow of lymph fluid. The lymphatic endothelial cells within the collecting vessels are interconnected by continuous, zipper-like junctions that provide tight cell–cell adhesions and prevent lymph leakage [19]. From collecting lymphatic vessels, lymph is transported into and out of lymph nodes through afferent and efferent lymphatic vessels, respectively. Ultimately, lymph drains to the bloodstream via the lymphaticovenous junction, where the major and minor thoracic ducts connect with the subclavian veins [16,20,21,22].

Generally, lymphatics are irregular in shape and collapse in histological sections, rendering histological visualization by hematoxylin-eosin-stained slides difficult. However, in recent years, many studies have identified lymphatic endothelial cells using immunohistochemical markers such as podoplanin, lymphatic vessel endothelial hyaluronic acid receptor-1 (LYVE1), prospero-related homeobox-1 (PROX-1), and vascular endothelial growth factor receptor-3 (VEGFR-3). Although none of these immunomarkers is entirely specific to lymphatic vessels, a panel including these markers may identify lymphatic endothelial cells [23,24,25].

An interesting example of the usefulness of immunohistochemical markers is the cornea. Indeed, the cornea was considered alymphatic but the use of novel lymphatic biomarkers has led to the detection of lymphatic endothelial cells [26]. Moreover, lymphatic vessels have been identified in the ciliary body. Indeed, immunohistochemical studies of post-mortem human eyes conducted by Yücel et al. revealed the presence of podoplanin- and LYVE1-positive lymphatic channels within the ciliary body stroma [27].

### 3.2. Ocular Lymphatics

Ocular lymphatics have recently been described in tissues such as corneal limbus, conjunctiva, extraocular muscles, eyelids and lacrimal glands [28]. A recent study demonstrated that limbal and conjunctival lymphatic distribution is markedly polarized toward the nasal side of the eye [11]. Recent research studies have shed light on the novel perspectives on ocular lymphatics. Schlemm’s canal is now recognized as a hybrid vessel exhibiting both vascular and lymphatic characteristics [27]. Schlemm’s canal (SC) is a specialized vascular channel lined with endothelial cells that perimetrically encompasses the corneal limbus. Its primary role is to drain aqueous humor back into the bloodstream via collector channels. As the main route for conventional aqueous humor outflow (AHO), Schlemm’s canal provides the necessary resistance to balance fluid production and drainage, which is critical for regulating intraocular pressure (IOP). Structural and functional similarities between Schlemm’s canal and lymphatic vessels have been observed [29,30], since this canal is lined with endothelial cells that resemble those of the lymphatic system. However, there are some differences between ocular lymphatics and Schlemm’s canal that deserve further analysis. For instance, limbal and conjunctival lymphatics are relatively thin and highly branched, whereas Schlemm’s canal is structurally thicker and remains unbranched [11]. Additionally, lymphatic vessels contain numerous luminal valves exhibiting high Prox1 expression, whereas Schlemm’s canal lacks comparable valve structures and lymphatic endothelial cell markers such as LYVE-1 and podoplanin [31]. Anatomically, limbal lymphatics are situated on the outer surface of the limbus, whereas Schlemm’s canal is positioned on the inner limbal side, adjacent to the iris base. Regarding expression of immunohistochemical biomarkers, both the ocular lymphatics and Schlemm’s canal express integrin α9, CD31, VE-cadherin, collagen IV, Prox-1 and VEGFR3 [11,32] while LYVE1 expression is exclusive to the ocular lymphatics [11,31,33,34]. Furthermore, Prox1 is strongly expressed in both the inner and outer walls of Schlemm’s canal [1,31].

As far as the existence of lymphatics in the choroid is concerned, immunohistochemical studies detected LYVE-1-positive cells but co-localization with the macrophage marker CD68 indicates that these cells are rather macrophages than true lymphatic structures [35]. Some studies propose that these LYVE-1/CD68-positive macrophages might contribute to a lymphatic-like transition under inflammatory conditions [7]. Nonetheless, some studies continue to claim the existence of choroidal lymphatics, though these findings have not been universally supported [36,37].

The existence of lymphatics in extraocular muscles has been a debatable matter. Damasceno et al. [33] reported the presence of lymphatic vessels within the connective tissue of all extraocular muscles, whereas Philips et al. [34] found no such vessels in these muscles, except in the anterior region of the levator muscle. Despite this discrepancy, both studies confirmed the presence of T and B cells in all examined extraocular muscles. This controversy may be attributed to differences in tissue sampling locations, demographic factors such as age and race, or the possibility that lymphatic vessels are restricted to the connective tissue rather than the muscle itself.

Another proposed role of ocular lymphatics concerns their potential involvement in facilitating drug delivery. The subconjunctival space serves as an important pool for therapeutically administered subconjunctival medications, including antibiotics and steroids. Subconjunctival drug injections create a distinct type of bleb, within which the administered drug may either permeate the sclera to reach its target or be inefficiently cleared by the conjunctival lymphatic system. Therefore, the high density of lymphatic vessels in proximity to the bleb could have a negative impact on drug delivery, since rapid drainage and clearance via conjunctival lymphatic outflow could significantly reduce the drug’s bioavailability [11].

**Figure 1:**
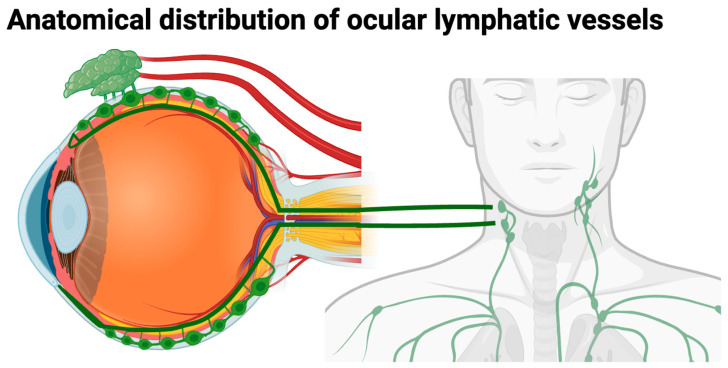
Anatomical distribution of presumed lymphatic vessels in ocular and periocular tissues based mainly on animal models. Lymphatic vessels are present in the conjunctiva, corneal limbus, ciliary body, lacrimal gland, and optic nerve sheath, extending through the optic nerve head toward the cervical lymph nodes in the neck. Presumed lymphatic vessels are depicted in green.

A diagrammatic representation of the localization of lymphatic vessels in ocular and periocular structures, based mainly on animal models, is shown in Figure 1. These vessels are found in the conjunctiva, corneoscleral limbus, ciliary body, lacrimal gland, and optic nerve sheath, whereas the central cornea and the retina are normally devoid of lymphatic vasculature under physiological conditions.

### 3.3. Aqueous Humor

Aqueous humor drainage from the anterior chamber is facilitated by ciliary body lymphatics. Aqueous Humor, a transparent, slightly alkaline fluid present in the anterior and posterior chambers of the eye, plays a crucial role in maintaining intraocular pressure and ocular shape while providing an optically clear medium for light transmission from the cornea to the retina [38]. Additionally, aqueous humor supplies nutrients and oxygen to avascular ocular tissues, such as the cornea, lens, and trabecular meshwork, and facilitates the removal of metabolic waste products from these tissues. Aqueous humor is continuously produced by the ciliary epithelium of the ciliary processes extending from the pars plicata, the anterior portion of the ciliary body [39]. The production rate is approximately 2.5 µL/min. This process occurs in three distinct phases. Firstly, blood is directed to the vascular network of the ciliary processes. Secondly, plasma undergoes filtration through the fenestrated ciliary capillaries into the ciliary stroma. Finally, the ciliary epithelium actively secretes the aqueous component of the plasma into the posterior chamber as aqueous humor [39,40]. The formation of aqueous humor is governed by three principal solute and fluid transport mechanisms: diffusion, ultrafiltration, and active transport. The secretion is facilitated by the active transport of ions and other molecules across the non-pigmented epithelium (NPE) of the posterior ciliary epithelium into the posterior chamber. This process generates an osmotic gradient across the NPE cells, thereby driving the passive movement of water into the posterior chamber through aquaporin (AQP) water channels, specifically AQP1 and AQP4, expressed by NPE cells [38,39,41]. Following its secretion into the posterior chamber, aqueous humor circulates around the lens and subsequently flows into the anterior chamber through the pupil.

Aqueous humor exits the eye through two distinct pathways: the conventional and the unconventional pathways. The conventional pathway, also known as the trabecular pathway, is the primary drainage route and involves the passage of aqueous humor through the trabecular meshwork, juxtacanalicular tissue, Schlemm’s canal, and collector channels, ultimately draining into the episcleral venous system. In the unconventional outflow pathway, aqueous humor traverses the ciliary muscle and subsequently passes through the supraciliary and suprachoroidal spaces [42]. From there, aqueous humor exits the eye via two principal routes: (1) the uveoscleral pathway, wherein aqueous humor permeates the sclera and drains into the orbital vasculature, or (2) the uveovortex pathway, in which aqueous humor enters the choroid and is subsequently drained through the vortex veins [43,44,45,46]. Furthermore, recent investigations have suggested the existence of a uveolymphatic pathway, wherein aqueous outflow is mediated by lymphatic vessels within the ciliary body. The presence of dilated lymphatic vessels within the bleb formed after trabeculectomy was first reported by van der Zypen et al. [47]. Although a direct lymphatic pathway in the ciliary body has been proposed [48], subsequent investigations have not consistently validated this finding [1,49]. Furthermore, evidence of lymphatic drainage following successful filtration surgery has been documented in individual studies [50,51]. An in vivo study conducted in rabbits and monkeys confirmed both the existence and functional significance of conjunctival lymphatics in facilitating aqueous humor drainage from conjunctival blebs post-filtration surgery [52]. More recently, a study in human subjects further validated lymphatic outflow from subconjunctival blebs through ocular surface lymphangiography and anterior segment OCT imaging in post-surgical patients [53]. It is important to highlight that the current literature does not clearly elucidate the differences in lymphatic involvement and environments, despite their notably distinct physiological conditions (e.g., pressure gradients, immune privilege, and tissue composition) [1,49]. The majority of suggested intraocular lymphatic or lymphatic-like mechanisms (e.g., those involving the ciliary body or Schlemm’s canal) remain rather indirect or theoretical, and they are primarily derived from experimental models [1,31]. On the contrary, extraocular compartments, particularly the conjunctiva, appear to have more consistent and functionally validated lymphatic networks, particularly in the context of filtration surgery [52,53]. Thus, although lymphatic participation in aqueous humor outflow has been proposed—especially under pathological or post-operative conditions—there is yet no definitive evidence to distinguish compartment-specific lymphatic roles. In this context, most of our current knowledge relies on data from developmental and experimental studies. Another study supported the concept that ocular lymphangiogenesis initiates with the emergence of nascent lymphatic vessels from the nasal side of the developing eye [11]. These lymphatics exhibit rapid growth, sprouting and bifurcating before encircling the cornea in both clockwise and counterclockwise directions toward the temporal side. It is a critical developmental phase since limbal and conjunctival lymphatics establish frequent interconnections until they encompass the entire conjunctival area. Remarkably, the nasal side consistently exhibits a higher density of lymphatic vessels compared to the temporal side, likely due to the primary entry of the major lymphatic trunk from the medial canthus region [11]. This polarized distribution of ocular lymphatics suggests that fluid drainage and immune surveillance may be more efficient in the nasal region than in the temporal region of the eye.

**Figure 2:**
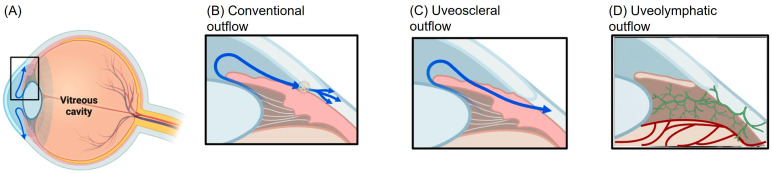
Aqueous humor outflow pathways and lymphatic contribution. Diagram illustrating aqueous humor production in the posterior chamber and its drainage through the anterior chamber via conventional, uveoscleral, and proposed uveolymphatic pathways. (**A**) Schematic representation of the posterior and anterior chambers. (**B**) Conventional outflow: Blue arrows indicate aqueous humor drainage through the trabecular meshwork and Schlemm’s canal. (**C**) Uveoscleral outflow: Blue arrows indicate unconventional drainage through the ciliary muscle and the supraciliary and suprachoroidal spaces, with uveoscleral and uveovortex routes. (**D**) Uveo-lymphatic outflow: Red lines indicate anterior segment blood vasculature, while green lines represent conjunctival lymphatic vessels forming the proposed lymphatic drainage pathway. Normal lymphangiogenesis.

A schematic illustration of aqueous humor formation and its drainage through both conventional and unconventional outflow routes is shown in Figure 2. The diagram also depicts the proposed uveolymphatic pathway involving conjunctival lymphatic vessels, which appears to play a particularly important role after filtration surgery.

It is generally accepted that the VEGF family plays a significant role in lymphangiogenesis. It consists of 5 members, i.e., VEGF-A, placenta growth factor (PlGF), VEGF-B, VEGF-C, and VEGF-D. Regarding VEGF-A, two mechanisms of action have been proposed: VEGFR-3-dependent and VEGFR-3-independent. Corneal lymphatic growth in response to VEGF-A occurs at a later stage than blood vessel formation, and requires higher VEGF-A concentrations for lymphangiogenesis compared to angiogenesis. The coordinated timing and spatial relationship between these processes suggest a functional interdependence between blood and lymphatic vessel development [54]. VEGF-D is also a ligand for VEGFR-2 and VEGFR-3 [55]. Another interesting mechanism of lymphangiogenesis involves macrophages via secretion of VEGF-A factor, which results in lymphangiogenesis as well as hemangiogenesis, exhibiting its action by binding to VEGFR-2 [56]. Conversely, macrophages release both VEGF-C and VEGF-D, which bind predominantly to VEGFR-3 [22,57]. Other factors such as FGF-A, HGF, PDGF and angiopoietin are also known lymphangiogenesis promoters. Remarkably, angiopoietin displays a crucial role in Schlemm’s canal formation and maintenance during adulthood [58]. A notable characteristic is that both HGF and FGF-induced corneal lymphangiogenesis can be blocked via inhibition of VEGFR-3 [59,60]. Lastly, IGF-1 and IGF-2 have been shown to significantly enhance the proliferation and migration of lymphatic endothelial cells, and therefore, corneal lymphangiogenesis. Importantly, IGF-1-mediated lymphatic vessel formation operates through a mechanism independent of VEGFR-3 signaling [61].

## 4. Ocular Glymphatic System

### 4.1. Normal Glymphatic System

In this review, we deemed it important to include recent studies highlighting the potential presence of an ocular glymphatic system, which, in conjunction with lymphatics, may have significant implications for retinal clearance and diseases impacting the posterior segment of the eye, i.e., the retina. The anatomical pathway of the glymphatic system comprises three key components: the para-arterial cerebrospinal fluid (CSF) influx route, the paravenous interstitial fluid (ISF) clearance route, and the transparenchymal pathway that relies on astroglial water transport via astroglial cells through the astrocytic aquaporin-4 (AQP4) water channel [62]. Aquaporin-4 is postulated to function as a key component of astrocyte endfeet, facilitating the exchange of cerebrospinal fluid (CSF) and brain interstitial fluid (ISF) through convective flow from para-arterial to paravenous spaces, driven by a pressure gradient generated by arterial pulsations. Such fluid transport plays a crucial role in clearing solutes and metabolic waste products from CSF and ISF, thereby maintaining brain homeostasis [63,64,65]. Emerging research suggests that an ocular glymphatic system may exist. [66]. Although no lymphatic drainage vasculature has been proven in the retina, the presence of an ocular glymphatic system may contribute to this clearance process. In recent years, researchers have proposed that a glymphatic system, akin to the one in the brain, plays a role in maintaining fluid homeostasis in the retinal layers [67,68]. Importantly, the retina, which forms the posterior segment of the eye, is viewed as a direct extension of the brain. Retinal ganglion cell axons extend beyond the retina, merging with optic nerve fibers to establish neural connections within the brain. In the central nervous system, the meningeal lymphatic vascular network is believed to interact with the brain’s glymphatic fluid transport system, which comprises paravascular spaces created by astrocytic endings enveloping the blood vessels of the blood–brain barrier [69]. By tracking the diffusion of intravitreously administered fluorescent amyloid-β as a tracer, Wang et al. provided evidence for a polarized clearance system in the posterior segment of the eye and along the optic nerve. The tracer was rapidly transported through the paravascular spaces of the optic nerve veins and was also detected in the cervical lymph nodes, indicating a potential role for the lymphatic system in this process [70]. Furthermore, in their review, Wostyn et al. analyzed cross-sections of human optic nerves using light microscopy following the bolus injection of India ink into the subarachnoid space of the optic nerve. The findings revealed the accumulation of India ink within the paravascular spaces surrounding the central retinal artery and vein, while the lumens of these vessels remained unstained. The ink deposits were observed between collagen fiber bundles, outlining a narrow, slit-like space [71].

### 4.2. Normal Glymphatic–Lymphatic Association

Although there is limited evidence of a substantial effect on intraocular pressure (IOP), the lymphatic system may play a role, in conjunction with the ocular glymphatic (glial lymphatic) system, in removing metabolic waste from the eye. It is described as follows: After its production, most of the aqueous humor exits the eye through the anterior outflow pathway. A smaller fraction, however, moves into the vitreous chamber, where intraocular pressure pushes it into the neural retina. Within the retina, aqueous humor mixes with interstitial fluid and travels along retinal ganglion cell axons, crossing the lamina cribrosa barrier [24]. It then exits the axons, moves toward the perivenous space, and ultimately drains into cervical lymph nodes via meningeal lymphatic vessels. Similarly, the optic nerve has its own glymphatic system. In this process, cerebrospinal fluid (CSF) from the subarachnoid space (SAS) enters the optic nerve parenchyma in parallel to the periarterial space. After undergoing glymphatic processing, CSF exits the optic nerve through the perivenous space and drains into cervical lymph nodes via meningeal lymphatics [24]. This could be particularly significant given the retina’s high metabolic activity, which produces various waste products, including neurotoxic proteins, that require clearance. It is still believed that the retina lacks a conventional lymphatic drainage system [14], but an eye glymphatic system may participate in this cleaning process. In the central nervous system, a meningeal lymphatic vascular system appears to be linked to a glymphatic fluid transport system within the brain parenchyma [69,72].

## 5. Lymphatics in Ocular Pathologies

**Pterygium**: A pterygium is a raised, superficial, and external ocular mass that typically originates from the perilimbal conjunctiva and extends onto the corneal surface. Pathologically, it is characterized by proliferative, invasive, and highly vascularized tissue [73]. Martín-López et al. (2019) found a threefold higher blood-to-lymphatic vessel ratio in pterygium compared to normal conjunctiva, emphasizing the significance of vascular network expansion in disease progression [74]. Fukuhara et al. examined VEGF-C expression in human pterygium [75] and its significance in disease mechanisms using Western blotting and immunohistochemistry. Their study compared VEGF-C and VEGFR-3 expression between pterygium and normal conjunctiva of humans. Lymphatic vessel density (LVD) as well as VEGF-C and VEGFR-3 expression were significantly higher in pterygium samples compared to normal ones. Moreover, they mention that VEGF-C levels correlate with LVS in the pterygial tissue [75]. Dong et al. (2016) identified TNF-α as a regulator of VEGF-C expression in conjunctival epithelial cells, suggesting its potential as a therapeutic target to inhibit lymphangiogenesis [76].

**Melanoma**: Sclera, which is devoid of lymphatic vessels, may be influenced under pathological conditions; hence, the sclera’s vascular privilege can be compromised. This is expected in cases of ciliary body melanoma with extraocular extension, which accounts for approximately 4% of uveal melanomas and is associated with a poor survival prognosis [77,78]. Hematogenous metastasis of ciliary body melanomas occurs rapidly, facilitating the formation of nascent lymphatic vessels due to the continuous contraction of the ciliary muscle and the extensive vascularization of the ciliary body [79]. It is also believed that lymphatics that encircle ciliary melanoma serve as prognostic factors in extraocular cases, while other research argues that nascent lymphatics can develop without extraocular expansion [80,81]. Heindl et al. have demonstrated the presence of nascent lymphatic vessels in ciliary body malignant melanomas with extraocular extension, whereas such vessels are absent in melanomas confined within the sclera [81,82].

Metastasis is driven either through hematogenous or lymphatic spread [81,83]. Lymphatic spread is associated with lymphatic proliferation, and specific lymphangiogenesis-related proteins expressed at conjunctival melanoma margins include: CXCL12, CXCR4, CCL21, and CCR7 [84]. Specifically, the lymphatic involvement in melanoma progression follows three possible mechanisms: (1) conjunctival lymphatics undergo proliferation and infiltrate the melanoma through the outgrowth of lymphatic capillaries, characterized by newly dividing lymphatic endothelial cells [85,86]; (2) melanoma cells invade and expand into peritumoral lymphatic vessels, promoting further lymphatic dissemination [78,85,87]; and lastly (3) a combination of both processes occurs [78]. Remarkably, lymphangiogenesis occurs early in the progression of precancerous intraepithelial lesions in conjunctival melanoma patients, and its augmentation coincides with the progression of precancerous lesions to invasive conjunctival melanoma [80]. Additionally, in another study, Heindl et al. observed that the emergence of precancerous lesions in conjunctival squamous cell carcinoma (SCC) is accompanied by the expansion of nascent lymphatic vessels, serving as a prognostic indicator for both metastasis and local recurrence [80,82]. It is worth noting that intraocular lymphatic vessels were identified in 60% of melanomas with extraocular extension, displaying a reticular architecture with multiple small lumina, distinct from the larger and more dilated lymphatic vessels observed in the periphery of the extraocular tumor component [77]. Lymphatic metastases may occur in uveal melanoma cases that involve secondary extra-scleral tumor extension [1]. It is also proposed that intraocular lymphangiogenesis may serve as a novel independent prognostic marker for uveal melanoma with extraocular extension, as it has been linked to a significantly higher risk of tumor-related mortality [1,88].

In vitro studies indicate that conjunctival and uveal melanoma cells express vascular endothelial growth factors VEGF-A, VEGF-C, and VEGF-D, along with their specific receptor VEGFR-3, which contribute to pro-lymphangiogenic functions [89].

**Glaucoma**: As mentioned above, the lymphatic system has been implicated in glaucoma pathogenesis, primarily through its role in aqueous humor drainage. Additionally, although dysfunction of the ciliary body lymphatics has been proposed as a contributing factor to glaucoma, there is currently no experimental evidence to substantiate this hypothesis.

Regarding glaucoma pathophysiology and its relevance to lymphatics, it remains unclear whether lymphatics are reduced in number or dysfunctional. If existing lymphatic vessels remain functional, pharmacological stimulation could be a suitable intervention. Conversely, if lymphatic vessels are either diminished or dysfunctional, the primary objective should be to induce lymphangiogenesis, followed by enhancing the function of the newly developed lymphatic network [42]. Additionally, Schlemm’s canal exhibits eminent structural and functional similarities to lymphatic vessels, sharing key molecular regulatory mechanisms with the lymphatic system. It is known that VEGF-C is essential for Schlemm’s canal development, as its absence impairs canal formation in murine and zebrafish models, as well as in human eye tissue [31,42]. Furthermore, the development and maintenance of Schlemm’s canal, along with atrial fluid efflux, depend on PROX-1 and Tie2 signaling, highlighting potential therapeutic targets for glaucoma treatment [90]. Recent research indicates that angiopoietin (ANGPT) growth factors are essential for lymphatic vessel development in the corneal limbus, and the loss of ANGPT1 and ANGPT2 in these vessels has been associated with glaucoma and ocular hypertension in mice [90]. Mutations in TEK (TIE2) and ANGPT1 have been correlated with glaucoma pathogenesis, emphasizing their critical role in Schlemm’s canal function. One study has shown that the Tie1 gene is highly expressed in both human and mouse Schlemm’s canal, and its deletion results in hypomorphic Schlemm’s canal formation and consecutively increased intraocular pressure (IOP) due to impaired canal development [91]. This highlights Tie1 as essential for Schlemm’s canal integrity and function, making it a promising therapeutic target for glaucoma and a potential candidate gene for the disease in humans [91]. Moreover, deletion of Angpt1/Angpt2 or Tie2 severely disrupts Schlemm’s canal integrity, leading to elevated IOP, retinal neuron damage, and impaired retinal ganglion cell (RGC) function.

**Diabetic Retinopathy**: As far as retina is concerned, there is no evidence of lymphangiogenesis under normal conditions; however, lymphatic transition has been proposed in diabetic retinopathy. Specifically, Loukovaara et al. identified lymphatic vessel–like structures in the posterior segment of human eyes affected by sight-threatening PDR [92]. Lymphatic endothelial markers, including VEGFR3 and Prox-1, along with partial evidence of LYVE-1, were detected in surgically excised neovascular specimens from PDR eyes [92]. While none of these markers is individually specific to lymphatic endothelial cells (LECs), their combined use allows for the differentiation of lymphatic vessels from the blood vasculature. It is suggested that ischemia- and inflammation-driven lymphatic-like vessels may contribute to proliferative diabetic retinopathy (PDR) in the human eye, since they observed aberrant vascular cell differentiation exhibiting a hybrid lymphatic and blood angiogenic phenotype. Their findings challenge the prevailing understanding of pathological vascular angiogenesis in PDR, indicating that, in addition to conventional blood vessels, lymphatic-like vessels may emerge either from pre-existing retinal vasculature, through lymphangiogenesis, or potentially via a combination of both mechanisms [92]. In addition, LYVE1+ cells have been characterized in the retina, though their function has remained undiscovered [35]. VEGFR-A is an extensively documented ocular neovascularization factor in diseases such as AMD and diabetic retinopathy.

**AMD**: In their work Nakao et al. examined samples from 2 uveitis and 4 AMD patients to study the presence of lymphatics in choroidal neovascularization (CNV). Immunochemistry was performed using LYVE-1 and podoplanin antibodies. Although LYVE-1(+) macrophages were found to infiltrate acute CNV, immunohistochemistry revealed no evidence of lymphatic vessel assembly. Clinical data from patient-derived CNV membranes similarly lacked LYVE-1/podoplanin-positive vasculature, indicating that lymphangiogenesis is absent in conditions such as AMD and uveitis [93]. A more recently published study by Cakir Ince et al. sought to investigate the relationship between AMD and lymphangiogenesis by quantitatively analyzing lymphangiogenesis biomarkers such as LYVE-1, PDPLN, VEGF-C and VEGFR-3, VEGFR-2 in human vitreous, aqueous and serum [94]. The findings of this study demonstrate a significant correlation between the impairment of lymphangiogenesis and the pathophysiology of Age-Related Macular Degeneration (AMD), particularly the wet type. Results show that patients with AMD exhibit significantly lower levels of specific lymphatic markers, including LYVE-1 in the vitreous and aqueous humor, as well as Podoplanin (PDPLN) in the vitreous and serum, compared to healthy controls. Conversely, levels of the growth factor VEGF-C are significantly elevated across all tested samples—serum, vitreous, and aqueous—in the AMD group. This increase is especially pronounced in wet AMD patients, where VEGF-C levels in the vitreous, aqueous, and serum are substantially higher than in controls. Despite the abundance of VEGF-C, its primary receptors, VEGFR-2 and VEGFR-3, fail to increase at a compensatory rate within ocular fluids. This imbalance results in significantly higher VEGF-C/VEGFR-2 and VEGF-C/VEGFR-3 ratios in the vitreous of AMD patients. The lack of a corresponding increase in VEGFR-3, which is essential for the formation of new lymphatic vessels, suggests that lymphangiogenesis is weakened or suppressed in AMD [94]. Ultimately, the study suggests that this weakened lymphatic clearance may play a critical role in the accumulation of fluid and inflammatory macromolecules characteristic of the disease.

**Herpetic stromal keratitis**: Herpetic stromal keratitis (HSK) constitutes a leading cause of unilateral blindness in developed countries. Herpetic eye disease manifests with ocular involvement, including keratoconjunctivitis, iridocyclitis [95], as well as epithelial, stromal, and endothelial keratitis [96], and acute retinal necrosis [96]. HSK has been found to induce hemangiogenesis and lymphagiogenesis. In a mouse model of HSV-induced keratitis lymphatic sprouting was observed using confocal microscopy utilizing LYVE-1 marker [97]. Remarkably, lymphatic vessels appeared as early as day one post-infection (PI) and continued to expand through day seven, because of animal mortality [97]. In comparison, animals that were mock-infected–scarified but treated with PBS instead of HSV-1–showed no lymphatic vessel infiltration into the cornea. This indicates that HSV-1 infection triggered corneal lymphangiogenesis [97]. Both vascular networks remained present even after the resolution of active viral replication [97]. A thorough understanding into the pathophysiology of HSK has been achieved through the elucidation of molecular mechanisms that induce corneal lymphangiogenesis. As stated above, the vascular endothelial growth factor (VEGF) family plays a crucial role in lymphangiogenesis. It includes five key members: VEGF-A, VEGF-B, VEGF-C, VEGF-D, and placenta growth factor (PlGF) [56,98,99]. Among these, VEGF-C and VEGF-D are particularly important in promoting lymphangiogenesis during bacterial infections and wound healing, whereas VEGF-A also exhibits pro-lymphangiogenic effects in vivo during inflammatory lymphangiogenesis, although it is considered less potent than VEGF-C and VEGF-D [56,100]. Research has indicated that activated macrophages are recruited to areas of inflammation, stimulating the production of pro-lymphangiogenic factors, which act as ligands for VEGFR-3, a known receptor expressed on lymphatic endothelial cells activating cell division and assisting the formation of new lymphatic vessels [56,101,102]. Furthermore, blocking the interaction between VEGF-C/D and VEGFR-3, as well as depleting macrophages, can reduce inflammatory lymphangiogenesis [103,104,105]. Wuest et al. identified VEGF-A as the only ligand capable of stimulating lymphangiogenesis in HSV-1 corneal infections. Notably, VEGF-A binding to VEGFR-2 results in the formation of lymphatic vessels that are more dilated and prone to leakage compared to those formed via VEGF-C/D and VEGFR-3 signaling [103,106,107]. These lymph vessels are less effective than those generated by VEGF-C or VEGF-D. Furthermore, VEGF-A levels peak within a day of infection -before leukocytes infiltrate the cornea- suggesting that HSV-1–infected cells are the primary source of VEGF-A. Additional sources of VEGF-A have been identified. In certain animals infected with HSV, leukocytes and macrophages play a role in VEGF production, since HSV-1 infection elevates VEGF-A levels by downregulating sVR-1 mRNA expression—a natural VEGF-A inhibitor—and promoting the degradation of intact sVR-1 proteins [108]. Specifically, matrix metalloproteinase-7 (MMP-7) facilitates this process by breaking down human sVEGFR-1, thereby increasing the local availability of VEGF for endothelial cells. By day 2 post-infection, as inflammatory cells infiltrate the corneal stroma, macrophages and neutrophils likely become additional contributors to VEGF-A production [109].

VEGF-A seems to be the key driver of lymphangiogenesis during the initial stages of HSV infection, while VEGF-C is mostly involved after immune mediators are recruited. Consequently, targeting both VEGF-A and VEGF-C in HSV-infected individuals could potentially reduce lymphangiogenesis and mitigate immune-related tissue damage. However, further research is required to confirm these findings [110]. Besides VEGF role, two cytokines have emerged as potential molecules that drive lymphangiogenesis. Human corneal epithelial cells infected with HSV-1 exhibit elevated production of the proinflammatory cytokines IL-6 and TNF-α [111], both of which promote lymphangiogenesis.

**Non herpetic keratitis**: Interestingly, apart from HSK, a variety of infectious keratitis conditions exhibit differing levels of association with lymphangiogenesis. It has been found that Resolvins D1 (RvD1) in diabetic Aspergillus fumigatus keratitis significantly reduced IL-8 and IL-6 levels, fungal burden, and ROS production by inhibiting the MAPK-NF-κB pathway while simultaneously enhancing vascularization and lymphangiogenesis [112]. Moreover, research on bacterial keratitis caused by *Pseudomonas aeruginosa* demonstrated that in later stages, the condition activates VEGF-C/VEGFR-3 signaling and macrophages, leading to corneal lymphangiogenesis. Interestingly, the study revealed that newly formed lymphatics in advanced bacterial keratitis aid in reducing edema and inflammation-induced corneal opacity, thus underpinning the beneficial role of lymphangiogenesis and suggesting that, despite its involvement in various pathological conditions, induced lymphangiogenesis could serve as a potential therapeutic approach for bacterial keratitis [112].

**Allergic conjunctivitis**: Allergic conjunctivitis (AC) constitutes a widespread condition, affecting between 20% and 40% of the population [113]. It can be categorized into various subtypes based on its duration, including acute, intermittent, and chronic forms [114]. Ocular allergies are characterized by an invasion of abundant immune cells such as mast cells, eosinophils, and Th2 lymphocytes [114]. Ocular allergies driven by type IV hypersensitivity present with prominent lymphangiogenesis, correlating with increased levels of VEGF-C, VEGF-D, and VEGFR-3 in mice [115]. Inhibiting VEGF receptors successfully alleviated clinical symptoms in mice [115]. Furthermore, studies indicate that Th2 cytokines such as IL-4, IL-5, and IL-13 promote lymphatic endothelial cell proliferation. Overall, a plethora of research studies [115,116,117] demonstrates that during the pathological changes in allergic ophthalmopathy, newly formed lymphatic vessels emerge in ocular tissues. Experiments inhibiting VEGFR have demonstrated a strong link between these nascent lymphatics and ocular allergic diseases. Consequently, suppressing the formation of new lymphatic vessels could help moderate the development and progression of allergic ophthalmopathies.

Experimental data on mice showed that corneal transplantation in allergic conjunctivitis leads to increased conjunctival inflammation and a faster ingress of inflammatory cells into the donor cornea, alongside earlier lymphangiogenesis, while previous research demonstrated that exposing the conjunctiva to allergens alone does not trigger corneal inflammation or lymphangiogenesis [118]. This suggests that increased perioperative corneal inflammation in allergic conjunctivitis occurs only when there is a direct inflammatory stimulus within the cornea, such as a suture [118].

**Dry eye disease (DED)**: Dry eye disease (DED) is a chronic ocular surface disorder causing irritation, pain, and visual impairment. It results from tear film hyperosmolarity or ocular surface damage and inflammation. Once considered a tear deficiency solely, DED is now recognized as a persistent inflammatory condition. A series of studies showed that under desiccating stress, the cornea developed lymphatic vessels without concurrent blood vessel growth [119]. Notably, corneal lymphangiogenesis was linked to the activation of CD11b+ dendritic cells (MHC-II+) in draining lymph nodes, suggesting that lymphangiogenesis in dry eye disease (DED) may play a role in facilitating adaptive immune responses [119]. Furthermore, corneal lymphangiogenesis in dry eye disease (DED) has been found to be driven by IL-17 secretion. This is supported by showing that topical application of an anti-IL-17 antibody effectively inhibited lymphangiogenesis, reduced infiltration of CD11b+ cells in the cornea, and ultimately alleviated the symptoms of DED [120]. Parallelly, a targeted suppression of key factors such as VEGF-C, IL-17, IL-1, and IL-1β could minimize or prevent lymphangiogenesis, thereby serving as an effective strategy to inhibit the progression of DED [82]. Moreover, in an experimental DED mouse model, knockdown of HIF-1α decreased mRNA expression of LYVE-1, VEGF-C, VEGF-D, and VEGFR-3, and reduced LYVE-1-stained lymphatic vessels in lacrimal glands [121]. This effect appears to involve the Dll4/Notch signaling pathway, though the exact mechanisms remain unclear [122]. It pinpoints that lymphangiogenesis during DED induction facilitates the resolution of DED-related inflammation by removing CD45+ cells from LGs [121].

Overall, it is deemed that corneal lymphangiogenesis occurs only in cases of severe ocular surface inflammation, while it is unlikely to be present in the early stages or mild-to-moderate forms of DED in humans. Thus, more rigorous experimental studies are necessary to further explore its role in DED [1].

**Sympathetic ophthalmia**: In the case of sympathetic ophthalmia, there is minimal research on its association with ocular lymphatics, and no definite correlation has been corroborated. Nevertheless, one study has suggested that in an experimental sympathetic ophthalmia model, subconjunctival administration of retinal S antigen in one eye triggered bilateral sympathetic uveitis, whereas direct intraocular injection failed to induce the condition [123]. A comparable process takes place during a penetrating injury accompanied by uveal tissue prolapse, allowing uveal antigens to encounter the lymphatic system [123,124].

Recently, experimental strategies targeting lymphangiogenesis have emerged to improve graft survival. Semaphorins, which serve as guidance cues for nerve axons, exhibit angiogenic properties. Notably, Semaphorin 3F eyedrop not only contributes to the angiogenic barrier of the retina, but it has also shown extended anti-lymphangiogenic effect in the cornea and enhanced graft survival in a murine high-risk keratoplasty model [125,126].

**Ocular Graft Versus Host Disease (GVHD)**: Ocular graft-versus-host disease (oGVHD) is a rapidly advancing autoimmune disorder that arises after hematopoietic stem cell transplantation. It generates intense ocular inflammation and disrupts the lacrimal functional unit, ultimately leading to severe, sight-threatening complications [125]. Apart from acute onset GVHD, chronic GVHD is frequent. Chronic ocular GVHD progresses rapidly, resulting in severe ocular surface disease characterized by autoimmune-mediated damage to the corneal and conjunctival epithelium, as well as the lacrimal gland. This leads to tear film deficiency and compositional alterations. The progression of the disease involves tissue dysfunction, pathological vascularization, and fibrosis, ultimately causing visual impairment or blindness [125]. In their research, Gehlsen et al. suggest that the cornea is a target tissue for GVHD-associated lymphangiogenesis, and that its extent correlates with the severity of systemic GVHD. In their study, corneal lymphangiogenesis was markedly increased 21–28 days following experimental bone marrow transplantation, coinciding with the transient expression of VEGF-C in the cornea. However, it remains unclear whether lymphangiogenesis arises solely as a consequence of GVHD pathophysiology or if it is additionally influenced by the conditioning regimen. Thus, more research is needed to elucidate the role of lymphatic vessels in GVHD.

Recently, various experimental strategies have emerged that target lymphangiogenesis to augment graft survival. Semaphorins, which are guidance cues for nerve axons, display angiogenic properties. Notably, Semaphorin 3F eyedrop not only contributes to the angiogenic barrier of the retina, but also extends the anti-lymphangiogenic effect in the cornea and enhances graft survival in a murine high-risk keratoplasty model [1,126]. Another noteworthy strategy, fine needle diathermy (FND), which is predominantly used as a method for corneal blood vessel regression, has also been reported as effective for corneal lymphatic vessel regression. Importantly, FND can improve corneal graft survival [127]. However, the use of FND alone may trigger the release of proangiogenic factors, potentially leading to unexpected effects such as corneal (lymph)angiogenesis. Le et al. report that supplemental anti-VEGF therapy—VEGFR1R2 Trap—significantly improves corneal lymphatic regression after FND surgery [128]. In their study, Dietrich et al. compared graft survival among different groups of corneal transplant recipients. Transplantation models were distributed in normal risk, hence avascular, in high risk, hence inflamed, and hematologic and lymphatic vascularized, in avascular high risk, hence inflamed and avascular, and lastly in alymphatic high risk recipient beds, hence inflamed and hematologic vascularized but alymphatic [9]. They concluded that corneal lymphatic vessels, rather than blood vessels, are the primary determinant of the high-risk status of a (murine) recipient bed.

The main ocular conditions associated with lymphatic involvement, along with their underlying biological processes, diagnostic tools, and clinical relevance, are summarized in Table 1.

## 6. Ocular Glymphatic System in Pathology

### 6.1. Glymphatic System

It is believed that dysfunction of the glymphatic system may play a role in impaired amyloid-β (Aβ) clearance, potentially contributing to the pathogenesis of Alzheimer’s disease [129]. Ιn a mouse model of Alzheimer’s disease (AD), it was shown that the clearance of tau protein, a key pathological component in neurodegenerative diseases, relies on AQP4-mediated fluid transport [130]. In 2015 research by Denniston and Keane theorized the presence of a paravascular transport mechanism in the retina and optic nerve, akin to the glymphatic system found in the brain, rendering it a possible candidate for the genesis of retinal diseases such as AMD [67]. Indeed, it has been proposed that the ocular glymphatic system may play a role in the development of AMD. This hypothesis is based on two aspects: (1) the glymphatic pathway is responsible for clearing metabolic waste, including amyloid beta peptide, which is known to accumulate in AMD and (2), glymphatic transport efficiency declines significantly with age, rendering it a major risk factor for AMD [131,132,133]. However, there is still no direct scientific evidence confirming these hypotheses [24].

Recently, new studies shed light on the possible role of the ocular glymphatic system in glaucoma pathogenesis. It is generally accepted that retinal ganglion cell degeneration—a hallmark of glaucoma pathology—derives not solely by hypertonia but also from drainage defects of neurotoxic substances [68]. Subsequently, during the following elimination process, the ocular glymphatic system is thought to establish a functional link with lymphatic vessels, with both systems playing a crucial role in preserving neuronal health within the eye.

### 6.2. Glymphatic-Lymphatic Association

Open-angle glaucoma has been postulated to be associated with amyloid clearance via the glymphatic pathway. Taking into consideration that amyloid-beta (Aβ) accumulation has been observed to rise in response to chronically elevated intraocular pressure (IOP) in animal models of experimentally induced ocular hypertension (OHT) and lead to retinal ganglion cell (RGC) degeneration, the identification of a paravascular clearance system within the eye constitutes a significant breakthrough [5,134,135]. This discovery may offer crucial insights into the pathophysiological mechanisms underlying primary open-angle glaucoma (POAG), potentially guiding the development of novel therapeutic approaches.

Based on magnetic resonance imaging (MRI) findings of Terson’s syndrome—characterized by vitreous hemorrhage associated with subarachnoid hemorrhage—Sakamoto et al. hypothesized the existence of an interconnected network of paravascular channels. These channels are thought to envelop the central retinal vessels within the optic nerve and extend into their retinal branches. Their proposed function is to facilitate fluid drainage from the subarachnoid space surrounding the optic nerve to the region beneath the internal limiting membrane, which demarcates the interface between the retina and the vitreous body [136]. In theory, a paravascular “retino-orbital” pathway, consisting of a para-arterial cerebrospinal fluid (CSF) influx route encircling the central retinal artery to reach the retina, followed by a para-venous clearance pathway around the central retinal vein, could aid in the removal of neurotoxic substances like amyloid-beta (Aβ) that accumulate as a result of increased intraocular pressure (IOP) [8,66,137].

In their experiment, Mathieu et al. [138] studied whether the CSF enters the optic nerve via a glymphatic pathway and whether this entry is size-dependent. Their investigation demonstrated the existence of a glymphatic route within the optic nerve, where cerebrospinal fluid (CSF) infiltrates the nerve parenchyma through paravascular channels. These spaces are notably delineated by AQP4-positive astrocytic endfeet, with the fluid transport mechanism exhibiting distinct size-dependency. Indeed, a novel size-dependent mechanism for molecular entry from the CSF into the optic nerve paravascular spaces was eventually identified, restricted by a 70 kDa cut-off. Because the majority of abundant CSF proteins—most notably the neuroprotective enzyme L-PGDS—are smaller than this threshold, they can readily penetrate the nerve parenchyma. The association between elevated L-PGDS concentrations and impaired CSF circulation in patients with normal-tension glaucoma and idiopathic intracranial hypertension suggests that paravascular flow obstruction and resulting protein sequestration may be central to optic nerve pathology [139]. These results underscore the importance of paravascular transport in maintaining optic nerve homeostasis.

### 6.3. Retina

Given that until now no definite answer has been given and that no retinal lymphatic system has been discovered, it is hypothesized that a glymphatic pathway system could exist in the retina and particularly at the macula, facilitating the clearance of waste solutes and proteins. Normally, the absence of a lymphatic network in the retina is compensated by the transepithelial fluid transport across the RPE. Of interest is the fact that at the optic nerve head, there appears to be a defect in blood–retina barrier integrity resulting in protein drainage from the retina. Impairment or imbalance of these clearance mechanisms may be associated with glaucoma, ocular surface inflammatory disorders, and other chronic eye diseases.

## 7. Therapeutic Options

Corticosteroids constitute a first-line treatment option for inflammatory corneal conditions and have been found to be potent inhibitors of lymphangiogenesis and hemangiogenesis [140]. A noticeable strategy, focused on regression of blood and lymphatic vessel formation in human corneas, includes the inhibition of insulin receptor substrate-1 (IRS-1), a cytosolic scaffolding protein that interacts with the VEGF-receptor complex. Aganirsen, which is an antisense nucleotide targeting IRS-1, has been proven effective in reducing hem- and lymphangiogenesis in vivo [141]. More importantly, it was shown that aganirsen inhibited hemangiogenesis at a dosage of 200 μM, whereas lymphangiogenesis inhibition commenced at a lower dose, at 100 μM [141]. This significant inhibition of corneal lymph vessels highlights the importance of IRS-1 on lymphatic inhibition, with an even more impactful role on downregulation of lymph vessel growth rather than blood vessel growth [141]. Aganirsen appears to act by directly interacting with lymphatic endothelial cells, thereby inhibiting their proliferation and VEGF-A expression. Apart from that, the IRS-1 blockade indirectly downregulates lymphangiogenesis by decreasing the expression of macrophage-derived growth factors, particularly VEGF-A and VEGF-C [141]. Moreover, administration of Aganirsen as eye drops has been successfully tested in phase II and III clinical trials and resulted in a significant decrease in corneal neovascularization in patients [142].

## 8. Potential Contribution of Bioinformatics in the Study of Ocular Lymphatics

Integrating state-of-the-art methods from interdisciplinary informatics into the study of ocular lymphatics could provide insights into the mechanisms underlying ocular lymphatics. Bioinformatics enables the analysis of biological data from high-throughput experiments, such as genomics, transcriptomics, and proteomics. For instance, single-cell RNA sequencing quantifies the expression of genes at the cellular level [143] and could be used to detect the expression of immunomarkers, such as LYVE1 and PROX1, in the cells of ocular tissues. There are already multiple publicly available resources containing raw sequencing data [144,145] that could facilitate this analysis. Comparative RNA sequencing between different states of ocular tissue is an effective strategy for revealing functions and relationships of genes associated with lymphatics. Such comparisons could be applied between healthy and diseased tissues [145,146] or wild-type tissues and tissues with partially (knockdown)/fully (knockout) suppressed expression of related genes. Such expression control can be achieved through gene editing based on the clustered regularly interspaced short palindromic repeats (CRISPR) [147]. Advancements in transcriptomics include experiments that yield gene expression data with spatial resolution. Based on spatial transcriptomics, one study presented evidence for the expression of LYVE1/VEGFR3 genes in the optic nerve sheath [148]. A recent advancement combines CRISPR-based gene expression perturbation with spatial transcriptomics, revealing interactions between human tumor and immune cells [149]. This experiment could be similarly leveraged for cells in the eye. As miRNAs regulate gene expression [150], expression profiling of the miRNAs that target biomarker genes could unravel RNA interference (RNAi)-based regulatory mechanisms of ocular lymphatics. Online bioinformatics databases could direct such investigations, as they include information for common miRNA targeting of relevant biomarker genes, such as LYVE1 and PROX1 [151], and which miRNAs are expressed in the eye [152]. Phylogenetic studies [153] could clarify the role of lymphatic-related genes in the human eye by observing evolutionarily conserved properties of ortholog genes across species.

The investigation of related proteins and biomolecular interactions is another important front for the study of lymphatic function in the eye. Analyzing the expression, sequences, and structures of involved proteins could yield information about their function and interactions. Proteomics experiments such as stable isotope labeling by amino acids in cell culture (SILAC) [154] or mass spectrometry (MS) [155] could identify biomarkers for lymphangiogenesis. Protein misfolding disrupts proteostasis and is linked to diseases in the eye [156]. The perturbation [157] of proteostasis, given the lymphatic system’s contribution to its maintenance [158], could aid in the study of ocular lymphatics. Beyond examining the properties of individual biomolecules, scrutinizing their interactions can lead to the characterization of biological pathways. This can be achieved through analysis of data from Förster resonance energy transfer (FRET) [159] experiments, which probe intermolecular interactions such as protein-protein interactions, or through the systematic evolution of ligands by exponential enrichment (SELEX) [160], which emphasizes protein-RNA interactions. Multi-omics approaches integrate data across different levels of a biological process and could provide a unified view of the ocular lymphatic function. Investigating gene [161] and protein sequences [162] of lymphatic endothelial factors in the eye can disambiguate their expression patterns and functional roles. Information on the participation of these biomolecules in biological pathways [163,164] beyond ocular lymphatics may provide higher-level insights into immune responses in the eye. Consulting cellular localization information [165] could lead to new spatially relevant experimental targets for the study of ocular lymphatics. The identification of conserved features through sequence similarity analyses [166] could reveal evolutionary relationships with ocular lymphatic-related mechanisms of other species. Protein–protein interaction [167] networks of key lymphatic markers, such as LYVE1, could uncover new mechanisms of eye-specific lymphatic activity. These data can be jointly used to formulate strong hypotheses and interpret experimental results.

Cheminformatics and deep learning approaches could be leveraged to study lymphatic function in the human eye. Molecular dynamics [168] is a prominent method in cheminformatics that allows cost-effective exploration of molecular conformations, particularly when experimental validation is not yet feasible. Due to ongoing progress over the last decades, hundreds of thousands of experimentally derived biomolecular structures are available in the Protein Data Bank [169], many of which are suitable for simulations. Simulating the conformational changes [170] in biomolecules or complexes that are crucial to ocular lymphatic pathways, could provide insights into pathway activation. Potential protein–protein interactions could be predicted via molecular docking computations [171], providing reliable targets for subsequent experimental validation. Quantitative structure-activity relationship (QSAR) models [172] could support investigations into how specific toxicities affect lymphatic function in the eye. These established models predict a compound’s biological activity from its chemical data. There are recent efforts to share proprietary QSAR models on a large scale, as a collaboration within the pharmaceutical industry [173], with the aid of machine learning to ensure confidentiality and privacy. Moreover, advancements in machine learning demonstrated greatly enhanced predictions with the advent of deep learning neural networks. Accurate predictions of biomolecular structures or complexes that are not yet experimentally determined, possibly due to great experimental challenges, have become available to the community [174,175,176]. For instance, there is no experimentally derived structure of LYVE-1 protein with full coverage in a public database [162], but there is a predicted structure available that suggests the existence of long flexible loops [174]. Deep learning could also facilitate the analysis of experimental data. For example, a recent study proposes a deep learning model that improves optical coherence tomography lymphangiography (OCTL) by imaging ocular lymphatic and aqueous vein vessels without contrast agents [177]. In general, existing computational methods offer significant opportunities for advancing ocular lymphatics research.

## 9. Key Challenges and Future Perspectives

Improving our understanding of the role of ocular lymphatics could reveal novel challenges, and exciting diagnostic and therapeutic perspectives. A critical area of interest is the link of lymphatic vessels to a wide spectrum of ophthalmic pathologies, including glaucoma, dry eye disease, and corneal transplantation. It appears that some elements of the conventional outflow pathway (e.g., Schlemm’s canal) have been found to express specific lymphatic markers such as Prox1 and VEGFR-3 but not podoplanin, indicating that lymphatic-related molecules might affect conventional aqueous humor drainage [31,32]. This observation emphasizes the value of quantifying the lymphatic, trabecular meshwork, and uveoscleral outflow pathways to enhance evaluation of the influence of pharmacological treatments on aqueous humor dynamics and intraocular pressure. It is preferable that these pathways be quantified by pre-clinical models at different time points, such as non-invasive techniques, i.e., near-infrared tracers and fluorescence lifetime imaging [178,179].

Regarding corneal diseases, research into lymphangiogenesis has yielded new possibilities for novel therapeutic approaches. For instance, treatments inhibiting [180,181] lymphangiogenesis, such as corneal cross-linking and anti-VEGFs like Bevacizumab, have been found to improve graft survival after high-risk corneal transplantation. However, significant challenges remain, particularly in understanding neovascularization and defining the long-term effects of these treatments on graft survival. Additionally, the involvement of lymphatic vessels in immune reactions and their potential to regulate corneal graft rejection require further investigation into disease-specific therapies, especially in high-risk transplant settings [181]. In disorders such as ocular graft-versus-host disease and dry eye disease, the role of lymphatic vessels remains unclear, suggesting that further studies are necessary to conclude whether the presence of lymphatic vessels is a cause or a consequence of these conditions [125].

Currently, the pathophysiological mechanisms underlying ophthalmic pathologies are still being explored, whereas the research on ocular lymphatics is limited. Due to the increasing need for tailor-made treatments to improve therapeutic outcomes for eye diseases, studies highlighted in this paper emphasize the pivotal role of ocular lymphatics in various eye conditions. It is anticipated that further research focusing on the link between lymphangiogenesis and ocular diseases will shed light on the pathogenic mechanisms, onset, progression, and therapeutic targets associated with lymphangiogenesis [31,58,112]. For instance, in glaucoma, the conventional outflow pathway (i.e., Schlemm’s canal) presents structural and functional similarities to lymphatic vessels, raising questions about its exact role in the regulation of intraocular pressure and the potential therapeutic effects of lymphatic modulation [182]. However, it remains vague whether lymphatics are dysfunctional or reduced in glaucoma, and therefore further research is needed to assess their exact implications. Recent advances in non-invasive imaging techniques and nanotechnology will facilitate more thorough understanding of aqueous humor dynamics in glaucoma models allowing the quantification of changes in trabecular meshwork, uveoscleral, and lymphatic outflows [183]. If lymphatics remain normal and functional in glaucoma, pharmacological stimulation could be a potential therapeutic strategy. On the other hand, if lymphatics are dysfunctional, promoting lymphangiogenesis should be the main goal [184,185].

Also, most of the work is based on animal models, with comparably few clinical trials, meaning that future efforts can be aimed at the translation of current therapy into the clinical setting. The role of lymphatic vessels in clinical diseases such as uveitis and intraocular tumors remains important, due to their involvement in the regulation of the eye-immune system interaction [81,186]. In particular, the uveolymphatic pathway plays a substantial role in draining proteins and cell debris, and stimulating lymphatics could prevent the accumulation of these materials in conditions such as pseudoexfoliation and other open-angle glaucoma. An improved understanding of the underlying mechanisms and translating them into clinical practice could be beneficial for the optimal management of these ophthalmic disorders.

Exploring the interactions between lymphangiogenesis and other biological processes (e.g., angiogenesis, fibrosis, and metabolism) will further clarify the complex interrelationships among various systems and processes in both health and disease [183]. Furthermore, advancing imaging technologies is a growing field of research, which is expected to enhance the in vivo visualization of lymphatic vessels and lymphangiogenesis with greater specificity and resolution [187]. This pursuit of high-resolution visualization is particularly critical for specialized structures like Schlemm’s canal, which exhibits a hybrid phenotype sharing molecular and structural characteristics with both the lymphatic and blood vascular systems [188].

The future of ocular therapy may involve both tissue-specific and pharmacological targeting of lymphatic vessels. Thus, the eye could become the first organ for which therapies modulating lymphangiogenesis are approved in the clinic. Imaging, nanotechnology, and metabolic modulation of endothelial cells will further our understanding of ocular diseases associated with lymphangiogenesis.

## 10. Limitations and Translational Considerations

A significant limitation in the study of ocular lymphatics is the reliance on animal models, particularly rodents and rabbits. While these models have provided valuable mechanistic insights, important anatomical and physiological variations exist among different species. These include differences in the size of ocular tissues, intraocular pressure dynamics, and lymphatic distribution. As expected, these variations may impact on the extrapolation of findings to human physiology. Moreover, several proposed lymphatic or glymphatic pathways remain challenging to validate in vivo in humans due to current imaging limitations. Therefore, several concepts analyzed in this review—such as lymphatic involvement in aqueous humor drainage or glymphatic clearance in the retina—remain partially hypothetical suggesting the need for further validation with clinical studies. As a result, it is important to be cautious when translating experimental findings into clinical practice, and future research should prioritize human-based studies and the development of reliable, non-invasive diagnostic tools.

## 11. Conclusions

The research field of ocular lymphatics has made significant progress in recent years, unveiling novel insights into the mechanisms of lymphangiogenesis and its role in ophthalmic disorders. Although our understanding has improved—particularly regarding the ocular fluid drainage system and the role of lymphatics in regulating intraocular pressure and metabolic waste—it is yet to be defined how these mechanisms are implicated in glaucoma and retinal diseases. Studies exploring the lymphatic vessels and neovascularization have recognized valuable therapeutic targets, but further research is mandated to understand the specific mechanisms of neoplastic vessels in several ocular conditions. It is noteworthy that the eye, traditionally considered “alymphatic”, has become a promising site for discovering new therapeutic strategies that could revolutionize the treatment of ophthalmic pathologies. The investigation of ocular lymphatic and glymphatic systems provides a new frontier for fluid homeostasis and metabolic waste clearance, with potentially significant reverberation in visual health. Developing novel drugs and therapeutic strategies targeting lymphangiogenesis could reform therapeutic approaches, offering improved outcomes to patients with previously limited treatment options.

## Figures and Tables

**Table 1:** Diagnostic approaches to ocular lymphatic-related processes.

Condition	Lymphatic Process	Diagnostic Tool	Evidence Level	Clinical Relevance
Glaucoma	Aqueous outflow/SC function	OCT/AS-OCT	Human + animal	IOP control
Filtering surgery	Bleb lymphatic drainage	Lymphangiography, OCT	Human	Surgical success
Corneal graft	Lymphangiogenesis	IVCM, markers	Mostly animal	Rejection risk
Ocular surface information	Lymphatic expansion	Experimental imaging	Animal > human	Disease severity

Summary of major ocular conditions associated with lymphatic involvement, including the underlying biological process, currently available diagnostic tools, level of supporting evidence (human vs. animal studies), and clinical relevance. SC: Schlemm’s canal; OCT: optical coherence tomography; AS-OCT: anterior segment optical coherence tomography; IVCM: in vivo confocal microscopy.

## Data Availability

No new data were created or analysed in this study. Data sharing is not applicable.
